# Existing and Potential Therapeutic Strategies for Lowering Lipoprotein(a) Levels: An Update

**DOI:** 10.3390/jcm15062179

**Published:** 2026-03-12

**Authors:** Igor Domański, Aleksandra Kozieł, Jurand Domański, Małgorzata Trocha

**Affiliations:** 1 Clinical Department of Diabetology, Hypertension and Internal Diseases, Wroclaw Medical University, 50-556 Wroclaw, Poland; 2 Department of Infectious Diseases, Liver Disease and Acquired Immune Deficiencies, Wroclaw Medical University, 50-367 Wroclaw, Poland; 3 Department of Preclinical Sciences, Pharmacology and Medical Diagnostics, Wroclaw University of Science and Technology, 58-376 Wroclaw, Poland

**Keywords:** lipoprotein(a), cardiovascular risk, lipid metabolism

## Abstract

Lipoprotein(a) [Lp(a)] is a low-density lipoprotein-like particle that contains a unique apolipoprotein(a) [apo(a)] component covalently bound to apolipoprotein B-100. Elevated levels of Lp(a) have been identified as a well-established and genetically determined risk factor for atherosclerotic cardiovascular disease, including coronary artery disease, stroke, and calcific aortic valve stenosis. In contrast to other lipids, Lp(a) concentrations are minimally influenced by lifestyle or traditional lipid-lowering therapies, emphasizing the necessity for novel treatment approaches. This narrative review summarizes current and emerging therapeutic strategies for reducing Lp(a) levels. Such strategies include traditional agents such as niacin and PCSK9 inhibitors, as well as innovative therapies such as antisense oligonucleotides, RNA interference-based molecules, and small-molecule inhibitors. The mechanisms of action of these agents, in addition to clinical trial data and their capacity to modify cardiovascular outcomes, are explored in further detail. Furthermore, the current status of clinical guidelines and the evolving role of Lp(a)-targeted therapies in cardiovascular risk stratification are reviewed. A particular emphasis is placed on therapies that are in the advanced stages of clinical development. These include late-phase outcome trials and orally administered agents, which have the potential to significantly impact future clinical practice. The integration of mechanistic data with ongoing and completed clinical studies has been undertaken in order to provide a comprehensive framework for understanding the therapeutic potential of Lp(a) in the context of cardiovascular prevention.

## 1. Introduction

Lipoprotein(a) [Lp(a)] is a lipoprotein particle structurally related to low-density lipoprotein (LDL), but characterized by the presence of an additional apolipoprotein(a) [apo(a)] component, covalently linked to apolipoprotein B-100 via a disulfide bond [1]. Apo(a) exhibits significant structural homology to plasminogen, suggesting potential interference with fibrinolytic activity. Since its discovery over half a century ago, Lp(a) has attracted considerable interest due to its strong, genetically determined association with atherosclerotic cardiovascular disease (ASCVD), independent of traditional lipid risk factors.

Plasma concentrations of Lp(a) are highly heritable and primarily determined by the number of kringle IV type 2 repeats in the Lp(a) gene. These levels are remarkably stable throughout life, largely uninfluenced by age, sex, diet, or physical activity, and vary more than 1000-fold between individuals. Elevated Lp(a) concentrations—particularly those above 50 mg/dL—have been strongly linked to an increased risk of myocardial infarction, ischemic stroke, peripheral artery disease, heart failure, and aortic valve stenosis. The high prevalence of elevated Lp(a) in the general population, combined with the lack of effective conventional treatment options, has made it a major target of interest in lipidology and cardiovascular prevention [2].

There is a lot of evidence from studies of people that supports the idea that high levels of Lp(a) cause atherosclerosis, stroke and problems with the valves in the heart.

A 2024 analysis from the Women’s Health Study (28,000 initially healthy U.S. women, 30-year follow-up) showed that those with the highest baseline Lp(a) levels had an adjusted hazard ratio of 1.33 (95% CI, 1.21–1.47) for first major cardiovascular events compared with those in the lowest quintile, independent of hsCRP and LDL-C. This demonstrates that a single mid-life Lp(a) measurement can provide long-term prognostic information.

Another study from 2025 showed that consistent secondary-prevention data in over 270,000 patients with established ASCVD demonstrate a continuous increase in recurrent ASCVD risk with higher Lp(a) levels. Furthermore, Lp(a) contributes to CAVS progression by depositing oxidized phospholipids and promoting valvular inflammation and calcification.

Despite decades of investigation, the physiological role of Lp(a) remains incompletely understood. However, increasing evidence supports its pathological role in atherogenesis, thrombosis, and inflammation. This review explores current understanding of Lp(a) biology and evaluates both established and emerging therapies aimed at reducing its plasma concentration, with particular attention to the clinical trial evidence supporting these interventions.

## 2. Search and Selection Approach

For this narrative review, a structured yet exploratory literature search was conducted to identify publications relevant to therapeutic strategies for lowering Lp(a). The search was performed in the PubMed/MEDLINE, Embase, and the Cochrane Library databases. In addition, clinical trial registries such as ClinicalTrials.gov and the EU Clinical Trials Register were consulted to gain insight into ongoing and recently completed studies. The last search was conducted on 5 January 2026, and the time frame covered all available records from database inception to that date.

The search strategy employed a flexible combination of free-text terms and controlled vocabulary (MeSH/Emtree), with Boolean operators used to capture key concepts related to available and emerging lipoprotein(a)-lowering therapies. Search terms included “lipoprotein(a)” or “Lp(a)”, along with terms related to treatment and management, such as “therapy” and “pharmacological intervention”, as well as specific drug classes and agents, including PCSK9 inhibitors (alirocumab, evolocumab), antisense oligonucleotides (pelacarsen), small interfering RNA therapies (olpasiran, lepodisiran), statins (including pitavastatin), lipoprotein apheresis, CETP inhibitors (obicetrapib), evinacumab, mipomersen, and niacin.

Applied filters included language (English and Polish) and study type, prioritizing human studies. Eligible publications comprised randomized controlled trials, phase 1–3 clinical trials, post hoc analyses of large cardiovascular outcome trials, observational studies of clinical relevance, and selected high-quality review articles providing mechanistic or contextual insights. Animal and purely in vitro studies were excluded unless directly relevant to understanding therapeutic mechanisms. Case reports, editorials, and conference abstracts without full data were excluded.

Study selection was guided by predefined inclusion criteria: relevance to Lp(a)-lowering interventions, reporting quantitative effects on Lp(a) concentrations, and/or evaluation of clinical outcomes or safety. When multiple publications addressed the same intervention, priority was given to the most recent, comprehensive, or methodologically robust reports. Reference lists of key articles were manually screened to identify additional relevant studies.

A simplified PRISMA-like selection process was applied. In total, 642 records were identified across databases and trial registries. After removal of duplicates and screening of titles and abstracts, 238 publications were assessed in full text, of which 99 studies were included in the final narrative synthesis.

To enhance reproducibility and transparency, a representative example of the complete search strategy used in PubMed/MEDLINE is provided below:

(“lipoprotein(a) alirocumab”)

Filters applied: Clinical Study, Clinical Trial; Humans; English OR Polish; all publication years up to 5 January 2026.

## 3. Physiology and Pathophysiology of Lipoprotein(a)

Lipoprotein(a) is a genetically inherited lipoprotein particle resembling low-density lipoprotein (LDL) in structure, but distinguished by the presence of an apo(a) that is covalently linked to apolipoprotein B-100. Lp(a) is composed of an LDL-like core containing cholesterol and triglycerides, to which apo(a)—a highly glycosylated protein homologous to plasminogen—is covalently bound. Apo(a) is synthesized in hepatocytes and secreted into the circulation, where it forms a covalent disulfide bond with apolipoprotein B-100 on LDL particles, giving rise to the mature Lp(a) particle [3,4]. The number of kringle IV type 2 repeats within apo(a) varies significantly between individuals, influencing both the molecular size of the protein and plasma Lp(a) concentration. Individuals with fewer kringle repeats typically exhibit higher circulating levels [5].

The precise physiological role of Lp(a) remains unclear, but the structural similarity between apo(a) and plasminogen suggests that they may have similar functions. Apo(a) can compete with plasminogen for binding to fibrin and endothelial cells, with the potential to inhibit fibrinolysis and modulate clot stability [6,7]. Experimental studies demonstrate that Lp(a)/apo(a) reduces plasminogen activation on fibrin and cell surfaces, upregulates endothelial PAI-1 expression, and attenuates thrombolysis in vivo. In transgenic mice expressing apo(a), enhanced thrombus formation has been observed after vascular injury [8,9]. However, human interventional data are less consistent: despite profound (~70–90%) reductions in Lp(a) with antisense oligonucleotide therapy, no improvement in clot lysis time or fibrinolytic markers was observed [10]. Large-scale prospective studies and Mendelian randomization analyses also failed to show a consistent association between Lp(a) variants and venous thromboembolism risk, with the majority of evidence supporting a lack of association [11].

Beyond its effects on fibrinolysis, Lp(a) acts as a major carrier of oxidized phospholipids (OxPLs) in human plasma. These OxPLs exert pro-inflammatory effects, including monocyte priming, sustained arterial wall inflammation, and metabolic reprogramming of immune cells. OxPLs such as oxPAPC drive macrophages toward a pro-inflammatory “Mox” phenotype via redox-sensitive signaling pathways [12], while apo(a)-bound OxPLs enhance glycolysis in endothelial cells, increasing GLUT1 expression, prostaglandin E_2_ production, vascular permeability, and monocyte transmigration. These mechanisms place Lp(a) at the interface of thrombosis, inflammation, and vascular remodeling [13,14,15]. Collectively, these findings underscore Lp(a) as a key mediator linking oxidative stress, vascular inflammation, and thrombosis.

**Figure 1:**
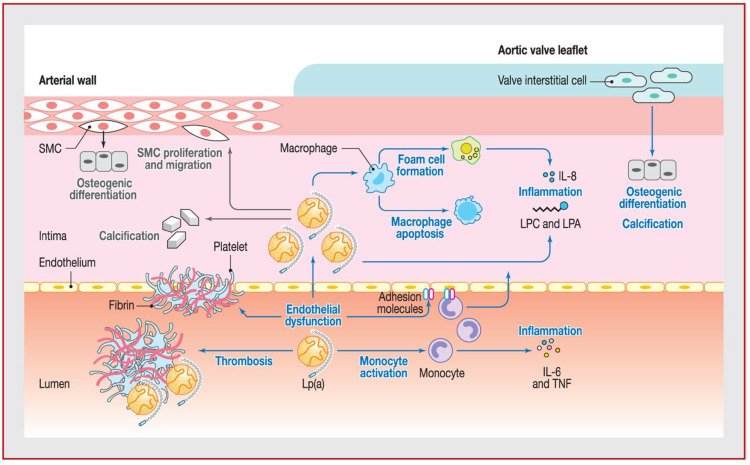
Cellular effects of Lp(a) and Lp(a)-associated oxidized phospholipids [19].

There exists consistent experimental and epidemiological evidence in support of the hypothesis that Lp(a) plays a proatherogenic role. The consequences of this process include the development of atherosclerotic plaque, vascular calcification, and thrombosis (Figure 1). These effects are independent of, and additive to, LDL cholesterol. Elevated levels of lipoprotein-associated phospholipids (Lp(a)) have been demonstrated to be strongly associated with calcific aortic valve disease. This association is supported by experimental and observational studies, which have shown that Lp(a)-derived oxysterols (OxPLs) can induce osteogenic and inflammatory signaling pathways, including RUNX2, BMP2, IL-6 and IL-8. In addition, Lp(a)-derived OxPLs have been shown to be able to predict faster progression of valve calcification on imaging follow-up [16,17,18].

**Figure 2:**
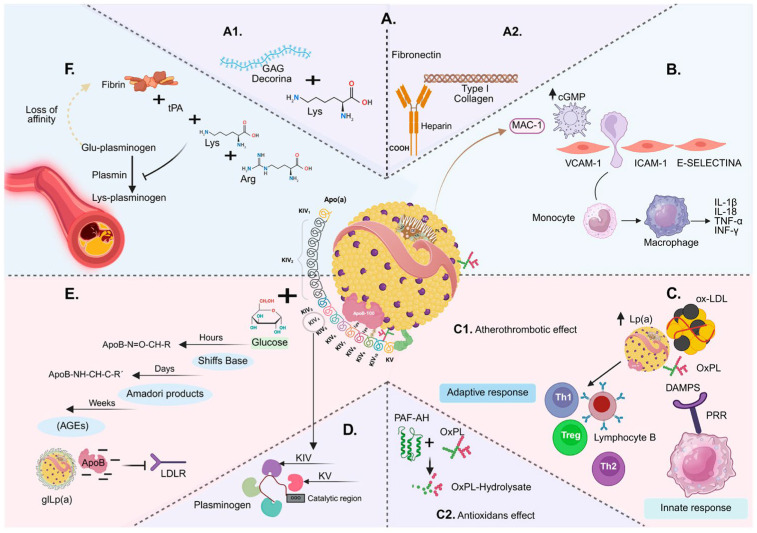
Model of Lp(a) pathophysiology in diabetes mellitus: (**A**) Endothelial and extracellular matrix anchoring: (**A1**) Apo(a) binds decorin/GAGs via lysine-binding sites. (**A2**) Subendothelial anchoring is further facilitated by Lp(a) interactions with fibronectin and type I collagen (heparin-facilitated), potentially involving MAC-1. (**B**) Endothelial activation and chemotaxis: Upregulation of VCAM-1, ICAM-1, and E-selectin promotes monocyte recruitment and differentiation into macrophages, leading to the release of pro-inflammatory cytokines including IL-1β, IL-18, TNF-α, and IFN-γ. (**C**) Lp(a)-bound oxidized phospholipids (oxPL): Elevated Lp(a) and associated oxPL act as DAMPs that activate PRRs, stimulating innate responses and modulating adaptive immunity: (**C1**) atherothrombotic effects and (**C2**) antioxidant activity, as PAF-AH on Lp(a) hydrolyses oxPL. (**D**) Lp(a) shows structural homology to plasminogen. KIV/KV domains in apo(a) compete for lysine-binding sites, impairing fibrinolysis (antifibrinolytic effect). (**E**) Non-enzymatic glycation of Lp(a): Glucose forms Schiff bases (hours) and Amadori products (days), progressing to AGEs (weeks). Glycation of apoB-100 reduces LDLR binding, favouring glycated Lp(a) [gLp(a)]. (**F**) Further atherothrombotic/antifibrinolytic effects: Occupation of lysine sites by Lp(a)/apo(a) reduces plasminogen binding and tPA activation, lowering fibrin affinity and promoting thrombo-resistance. Adapted from: [22], Created in BioRender. Osorio, E. (2025). Sequential model of Lp(a) pathophysiology in diabetes mellitus. https://BioRender.com/uenhksj.

Finally, the relationship between Lp(a) and glucose metabolism has been explored in large cohort studies. Interestingly, very low Lp(a) concentrations have been associated with an elevated risk of type 2 diabetes, insulin resistance, and hyperinsulinemia [20,21]. A proposed mechanism involves enhanced hepatic triglyceride synthesis and impaired VLDL clearance in the setting of genetically low Lp(a), leading to ectopic lipid accumulation in the liver and muscle and subsequent deterioration of insulin signaling [21]. In contrast, in established diabetes, Lp(a) retention in the arterial intima through glycosaminoglycans, proteoglycans, and fibronectin promotes early vascular inflammation. Retained Lp(a) activates endothelial signaling, upregulating adhesion molecules (VCAM-1, ICAM-1, E-selectin) and guiding monocyte chemotaxis and differentiation into macrophages, which produce proinflammatory cytokines (IL-1β, TNF-α) and reactive oxygen species, creating a proatherogenic microenvironment. Persistent hyperglycemia further modifies Lp(a) via non-enzymatic glycation, altering its charge and clearance, promoting accumulation in the arterial wall, foam cell formation, and activation of the AGE-RAGE axis, which amplifies oxidative stress, inflammation, and endothelial dysfunction (Figure 2) [22].

However, it should be noted that the associations observed are of an observational nature and thus do not allow for the establishment of causal relationships.

In the absence of approved Lp(a)-specific therapies, early and intensive management of other modifiable cardiovascular risk factors remains essential. This includes rigorous regulation of LDL cholesterol, blood pressure, glycemic status, and lifestyle-related risk factors. However, there is compelling evidence indicating a correlation between elevated Lp(a) levels and atherosclerotic cardiovascular disease, as well as calcific aortic valve disease. This underscores the pressing need for the development of targeted therapeutic interventions that either directly reduce Lp(a) synthesis or enhance its clearance, with the aim of reducing cardiovascular risk.

## 4. Available Therapeutic Strategies

### 4.1. Statins

Although some statins—particularly atorvastatin—can modestly increase Lp(a) levels through upregulation of hepatic Lp(a) mRNA and apo(a) synthesis, these changes are small and do not negate the substantial reductions in atherosclerotic cardiovascular events achieved with statin therapy [23]. In fact, multiple cohort studies and clinical trials have shown that in patients with elevated Lp(a), the cardiovascular benefits of LDL-C lowering with statins far outweigh any potential risk related to a modest Lp(a) increase [24]. Therefore, discontinuation of statins solely because of elevated Lp(a) is not warranted. Nonetheless, statin selection may be guided by potential differences in their effect on Lp(a), as emerging evidence and meta-analyses suggest that pitavastatin tends to lower Lp(a) or cause smaller increases compared with atorvastatin [25]. The proposed mechanism underlying this more favorable profile includes reduced induction of apo(a) synthesis as well as pleiotropic metabolic effects such as increased adiponectin levels, which may exert anti-inflammatory and potentially Lp(a)-modulating actions [26].

What we know: Statins are considered the mainstay of cardiovascular risk reduction, with substantial and consistent evidence supporting their efficacy in reducing major adverse cardiovascular events (MACE) through LDL-C lowering. The effect of these drugs on Lp(a) is reported to be either neutral or modestly increasing, and is not considered to be of clinical significance.

What remains uncertain: The question of whether increases in Lp(a) induced by statin therapy possess independent long-term prognostic significance remains unresolved. Nevertheless, the current body of evidence does not support the modification of statin therapy based solely on Lp(a) levels.

### 4.2. Ezetimibe

Ezetimibe is a selective inhibitor of the Niemann-Pick C1-like 1 (NPC1L1) transporter protein. The majority of available evidence indicates that ezetimibe has no significant impact on Lp(a) concentrations [27,28]. Importantly, studies have not demonstrated any clinically meaningful increase in Lp(a) levels with ezetimibe use. In such cases, ezetimibe may be considered as part of a combination therapy strategy, enhancing lipid-lowering efficacy without adversely affecting Lp(a).

Nonetheless, some analyses suggest a potential modest lowering effect of ezetimibe on Lp(a). A meta-analysis of trials evaluating 12 weeks of therapy demonstrated heterogeneous results, ranging from no effect to a reduction of up to 25%, with an overall mean decrease of 7.06% (*p* = 0.005) [29,30]. Proposed mechanisms include anti-inflammatory properties of ezetimibe, as well as secondary effects related to reductions in LDL-C and apolipoprotein B via upregulation of LDL receptors, which may indirectly contribute to lower Lp(a) concentrations [31,32]. However, it should be emphasized that despite this statistically significant reduction, the effect size is unlikely to be clinically relevant.

What we know: Ezetimibe has minimal and clinically negligible effects on Lp(a), with modest reductions reported inconsistently across studies. The cardiovascular benefits of statins are attributable to their ability to reduce low-density lipoprotein cholesterol (LDL-C).

What remains uncertain: The potential for specific patient subgroups to derive a modest Lp(a)-related benefit remains unclear, and current data do not support its use as an Lp(a)-targeted therapy.

### 4.3. PCSK9 Inhibitors

Monoclonal antibodies such as alirocumab and evolocumab inhibit the PCSK9 (proprotein convertase subtilisin/kexin type 9 inhibitors) protein, which is responsible for the degradation of low-density lipoprotein receptors (LDLR) on the surface of hepatocytes. This increases the clearance of LDL from plasma.

In addition to their LDL-C-lowering properties, PCSK9 inhibitors have been consistently shown to reduce Lp(a) concentrations by approximately 20–30%, as demonstrated in multiple randomized clinical trials [33].

As the FOURIER clinical trial shows, treatment with the PCSK9 inhibitor—evolocumab—resulted in a significant 26,9% reduction in Lp(a) within 48 weeks of therapy. Moreover, the absolute 26,9% reduction in Lp(a) occurred within 48 weeks of therapy. Additionally, the absolute reduction in Lp(a) was higher for individuals with higher baseline Lp(a) concentrations (up to 36 nnol/L) [34].

Another clinical trial, known as ODYSSEY OUTCOMES (where the PCSK9 inhibitor—alirocumab—was observed), showed a similar effect on the reduction in Lp(a) [35]. The higher the concentration of Lp(a) that was observed in serum, the greater the reduction that was noticed (median reduction −23%) [36].

While the precise mechanism by which PCSK9 inhibition lowers Lp(a) levels is not fully understood, recent findings suggest that these inhibitors may reduce Lp(a) levels in a few different ways. One mechanism has been demonstrated to lower plasma Lp(a) concentration by decreasing Lp(a) production [37].

Further research shows that, when combined with a statin, evolocumab may also increase Lp(a) catabolism by significantly upregulating the LDL receptor, thereby enhancing Lp(a) holoparticle clearance [38].

Furthermore, other investigations show that, in human hepatocytes and dermal fibroblasts, the secretion of apo(a) increases rapidly in the presence of the PCSK9 protein, and this effect is reversed in the presence of the PCSK9 inhibitor alirocumab [39].

PCSK9 inhibitors constitute a valuable therapeutic option—particularly in high-risk patients with elevated Lp(a) levels who do not achieve optimal lipid control with statins or other conventional agents.

What we know: PCSK9 inhibitors have been shown to reduce Lp(a) by approximately 20–30% in patients with higher baseline concentrations, with stronger absolute reductions observed in these cases. Large randomized trials have demonstrated significant cardiovascular risk reduction.

What remains uncertain: The extent to which cardiovascular benefit is directly mediated by Lp(a) lowering, as opposed to LDL-C reduction, remains incompletely defined.

### 4.4. Niacin

Niacin, by binding to the GPR109A receptor (also known as the niacin receptor), inhibits the activity of lipase in adipose tissue, leading to a reduction in the release of free fatty acids into the bloodstream. Administered in high doses, niacin exhibits pleiotropic effects on lipid metabolism, particularly concerning serum lipoprotein levels [40,41].

Clinical studies consistently demonstrate a significant reduction in Lp(a) levels with niacin therapy. Depending on the study population, Lp(a) concentrations may be reduced by approximately 20–40%, with reductions exceeding 60% observed in selected cases [42,43].

Precise mechanisms by which niacin affects Lp(a) metabolism still remain unclear. We hypothesized that apo(a) size can predict the niacin response in patients with elevated Lp(a) levels. There have been no previous studies evaluating extended-release (ER) niacin effects on Lp(a) level depending on apo(a) phenotype [44].

However, it is important to consider that niacin leads to an increased risk to develop new onset diabetes, bleeding, and infection risk [40,45]. Other side effects reported are mild increases in uric acid and liver enzymes, hypotension, nausea, vomiting, diarrhea, and precipitation of angina in patients on vasodilators [46,47].

Moreover, despite the fact that niacin has been demonstrated to significantly reduce levels of Lp(a), as evidenced by research studies, this does not correspond to a decrease in cardiovascular risk [40,48]. This combination of adverse effects, with the known existence of safer therapeutic alternatives, results in the limitation of the use of this substance.

What we know: Niacin has been demonstrated to reduce Lp(a) levels by 20–40%, with a greater effect observed in certain phenotypes.

What remains uncertain: Despite the significant lowering of Lp(a) that has been demonstrated, no cardiovascular outcome benefit has been shown, and safety concerns limit its clinical applicability.

### 4.5. Lipoprotein Apheresis

Studies have demonstrated that lipoprotein apheresis effectively reduces serum levels of Lp(a). Regular treatments performed every 1–2 weeks result in an approximate 25–40% reduction in Lp(a) per treatment session [49]; however, this effect is transient and does not accumulate over time [50].

Despite its benefits, lipoprotein apheresis is associated with several limitations that may hinder its widespread application. It is an invasive procedure. Each session may last several hours [51].

What we know: It has been demonstrated that lipoprotein apheresis produces substantial acute reductions in Lp(a), particularly in patients considered to be at extreme risk and who also have progressive ASCVD.

What remains uncertain: The evidence for this is largely observational, and the long-term magnitude of cardiovascular benefit attributable specifically to Lp(a) removal remains uncertain.

### 4.6. Mipomersen

Mipomersen is a second-generation antisense oligonucleotide that functions by inhibiting the synthesis of apolipoprotein B100. The molecular interaction between the drug molecule and the mRNA produced during APOB gene transcription instigates a process known as ribonuclease H-dependent degradation of mRNA. This, in turn, results in a consequent inhibition of gene expression. Mipomersen has been demonstrated to reduce blood levels of apolipoprotein B, total cholesterol, LDL and VLDL lipoproteins, triglycerides, and lipoprotein (a). The drug has been demonstrated to be highly effective in clinical studies, with studies showing a reduction in LDL-C concentrations of 20–50% and Lp(a) of 24–33% [52,53,54]. However, it should be noted that due to its high hepatotoxicity, the drug has not been approved for marketing in Europe by the European Medicines Agency [55], but is available in the United States [56].

What we know: Mipomersen has been demonstrated to reduce Lp(a) by approximately 25–30% and significantly lower apoB-containing lipoproteins.

What remains uncertain: The primary concerns that persist in this regard pertain to the potential for hepatotoxicity and safety issues, which have thus far impeded its wider clinical utilization and formal approval at the European level.

A summary of existing therapies for lowering Lp(a) levels is presented in Table 1.

## 5. Experimental Therapeutic Strategies

### 5.1. Small Interfering RNA

Small interfering RNA (siRNA) is defined as a short, double-stranded RNA molecule with a typical length of 20–25 nucleotides. It plays a crucial role in the natural regulatory process known as RNA interference (RNAi). This mechanism enables cells to regulate gene expression post-transcriptionally by selectively silencing specific genes. siRNA molecules are designed to recognize and bind to complementary sequences on messenger RNA (mRNA), which serves as a template for protein synthesis [57,58].

In the context of Lp(a), siRNAs are engineered to target the mRNA encoding apo(a). By binding with high precision to apo(a) mRNA, the siRNA guides a cellular machinery known as the RNA-induced silencing complex (RISC) to this transcript. The RISC then cleaves and degrades the targeted mRNA, thereby preventing its translation into the apo(a) protein [59].

Consequently, the synthesis of apo(a) in hepatocytes (liver cells) is significantly reduced, resulting in a decrease in the assembly and secretion of Lp(a) particles into the bloodstream. This process, when viewed over an extended period, has been shown to result in a significant decrease in plasma concentrations of Lp(a). siRNA-based therapies provide a highly specific and effective molecular approach to reducing Lp(a) levels by directly interfering with the genetic instructions responsible for its production [60,61,62].

Currently, numerous clinical trials are underway to investigate the therapeutic potential, efficacy, and safety profile of RNA-based therapies aimed at lowering Lp(a) (Table 2).

Among the most notable siRNA molecules being tested are Olpasiran, Zerlasiran, and Lepodisiran. Olpasiran is a small interfering RNA molecule that targets the expression of Lp(a), leading to the degradation of apo(a) mRNA and, consequently, preventing the assembly of Lp(a) particles in hepatocytes. Published results from Phase 2 and 3 clinical trials have shown promising effects. In the studied population, Lp(a) levels decreased by 70–90% by the 36th week of treatment [59]. Zerlasiran, currently in Phase 2 clinical trials, has also demonstrated significant Lp(a) reduction, with results indicating a decrease of more than 85%. This suggests its potential as a highly effective therapeutic option [63]. Lepodisiran has shown similarly high efficacy in reducing Lp(a) levels by 75–90%, while also demonstrating a high tolerance profile [64,65]. Ongoing studies are focusing on evaluating the long-term effectiveness of these therapies, as well as their safety and tolerance in broader populations. The most important ongoing clinical trials are: ACCLAIM-Lp(a)—Phase 3 on Lepodisiran—assessing the efficacy of Lepodisiran in reducing cardiovascular risk [66]; OCEAN(a) Outcomes Phase 3 on Olpasiran—the Impact of Olpasiran on Major Cardiovascular Events in Participants with Atherosclerotic Cardiovascular Disease and Elevated Lipoprotein(a). For Zerlasiran, the most important trial is a Phase 2 trial called ALPACAR-360—it evaluates efficacy, safety, and tolerability [67].

It seems that the promising results from these siRNA-based therapies could revolutionize the management of cardiovascular diseases associated with elevated Lp(a) levels.

Currently registered active clinical trials involving small interfering RNAs, along with their objectives and interventions, are summarized in Table 2.

**Table 2:** Clinical trials investigating RNA-based therapies for lowering Lp(a).

Aim of Trial	Conditions	Interventions	Control	Enrollment	Phases	Trial Status	NCT Number	Reference
Evaluate safety, tolerability, PK and effect on Lp(a) levels	Healthy	Lepodisiran	Placebo	66	Phase 1	Completed	NCT04914546	[68]
Assess PK of two LY3819469 formulations, safety, and tolerability	Healthy	Lepodisiran	-	27	Phase 1	Completed	NCT05932446	[69]
Evaluate efficacy and safety of LY3819469 in adults with high Lp(a)	Lipoprotein Disorder	Lepodisiran	Placebo	320	Phase 2	Completed	NCT05565742	[70]
Assess PK and safety in renal impairment vs. normal renal function	Renal Insufficiency Healthy	Lepodisiran	-	26	Phase 1	Completed	NCT05841277	[71]
Evaluate PK and tolerability of Lepodisiran in liver impairment	Liver Dysfunction Healthy	Lepodisiran	-	33	Phase 1	Recruiting	NCT06916078	[72]
Assess efficacy of Lepodisiran in reducing cardiovascular risk	ASCVD Elevated Lp(a)	Lepodisiran	Placebo	16,700	Phase 3	Recruiting	NCT06292013	[66]
SC Olpasiran vs. placebo: % change in Lp(a): Evaluate % change in Lp(a) from baseline	Cardiovascular Disease	Olpasiran	Placebo	281	Phase 2	Completed	NCT04270760	[73]
Olpasiran vs. placebo: CHD death, MI, or revascularization in ASCVD: Compare event risk in ASCVD with high Lp(a)	ASCVD	Olpasiran	Placebo	7297	Phase 3	Active, not recruiting	NCT05581303	[74]
QTc interval study: single/supratherapeutic dose: Assess ΔΔQT/QTc after Olpasiran doses	Basic Science	Olpasiran Moxifloxacin	Placebo	32	Phase 1	Completed	NCT06411860	[75]
Evaluate PK of Olpasiran in Chinese population	Elevated Serum Lp(a)	Olpasiran	-	24	Phase 1	Completed	NCT04987320	[76]
Compare PK in hepatic impairment vs. normal	Hepatic Impairment	Olpasiran	-	25	Phase 1	Completed	NCT05481411	[77]
Compare PK in renal impairment vs. normal	Renal Impairment	Olpasiran	-	33	Phase 1	Completed	NCT05489614	[78]
Evaluate efficacy, safety, and tolerability of Zerlasiran vs. placebo	ASCVD Lp(a)	Zerlasiran	Placebo	180	Phase 2	Completed	NCT05537571	[67]
Investigate safety and tolerability of Zerlasiran in patients with elevated Lp(a)	Hyperlipidemias Dyslipidemias Elevated Lp(a)	Zerlasiran	Placebo	70	Phase 1	Completed	NCT04606602	[79]

### 5.2. Lp(a) Inhibitor

Another approach to therapies aimed at lowering Lp(a) levels in the blood involves the development of inhibitors of Lp(a) synthesis. Muvalaplin, a small-molecule inhibitor of Lp(a) formation, reduces circulating Lp(a) levels by binding to the KIV7–8 domains of apo(a) and preventing its interaction with apoB on LDL particles. This inhibition disrupts the assembly of Lp(a) particles in the liver, thus leading to a reduction in Lp(a) levels in circulation [80,81]. Muvalaplin is well-tolerated and demonstrates efficacy in reducing Lp(a) levels by 60–85% [82,83]. An important feature of this new investigational drug is its oral administration form, which provides a significant advantage.

Currently, multiple clinical trials are ongoing, both in healthy participants to evaluate the drug’s effectiveness and safety profile, and in patients with pre-existing health conditions, including those with kidney disease. This wider population of patients is critical, as Lp(a) levels are often elevated in individuals with chronic kidney disease. The most recent trial is a Phase 3 trial on Muvalaplin called MOVE-Lp(a) that evaluates the efficacy of the drug in reducing cardiovascular risk in participants with high lipoprotein(a) who have cardiovascular disease or are at risk of a heart attack or stroke [84].

Currently registered active clinical trials involving Lp(a) Inhibitors, along with their objectives and interventions, are summarized in Table 3.

### 5.3. Cholesteryl Ester Transfer Protein

Cholesteryl Ester Transfer Protein [CETP] is a plasma protein whose main function involves the exchange of cholesterol esters from HDL to triglycerides in VLDL and LDL. This transfer process influences the lipid composition of these lipoproteins, contributing to the regulation of cholesterol levels in the bloodstream and leading to increased levels of HDL cholesterol and decreased levels of LDL cholesterol and triglycerides [90,91]. Among third-generation CETP inhibitors, Obicetrapib is one of the leading candidates [92].

In addition to its proven effectiveness in lowering LDL levels [47], this compound reduces Lp(a) levels by more than 50% [48]

Clinical trials are currently underway to explore the further development of this drug in targeting Lp(a), as well as to evaluate its safety and efficacy.

Since there are many clinical trials underway on Obicetrapib, it is worth focusing on the most important ones—phase 3 trials. The first of these, known as TANDEM, aims to evaluate the efficacy, safety, and tolerability of obicetrapib in patients with heterozygous familial hypercholesterolemia (HeFH) and/or atherosclerotic cardiovascular disease (ASCVD) [93]. Another phase 3 clinical trial, known as PREVAIL, is a multicenter study on the efficacy of Obicetrapib treatment in patients with ASCVD who have not achieved their treatment goals with other lipid-lowering therapies despite using the maximum tolerated doses [94].

Currently registered active clinical trials involving Cholesteryl Ester Transfer Protein, along with their objectives and interventions, are summarized in Table 4.

### 5.4. Antisense Oligonucleotides

Antisense oligonucleotides (ASO) are short, synthetic strands of nucleic acids engineered to bind specifically to complementary sequences of target mRNA, thereby modulating gene expression—most commonly via RNase H-mediated degradation of the mRNA strand. This approach enables highly selective downregulation of pathogenic proteins at the transcriptional level.

In the case of Lp(a), one of the most promising ASO-based therapies under investigation is pelacarsen. This next-generation, GalNAc-conjugated ASO is specifically designed to target Lp(a) mRNA in hepatocytes, where apo(a) is synthesized, thereby effectively suppressing production of apo(a) at its source.

Clinical studies have demonstrated that pelacarsen produces robust and sustained reductions in plasma Lp(a) levels, in the range of approximately 30–70%, depending on dose and regimen. Notably, these effects have been achieved with a favorable safety and tolerability profile, with no major safety concerns or treatment-limiting adverse events observed to date [111,112,113].

Pelacarsen is currently undergoing evaluation in multiple ongoing clinical trials, including the large-scale Phase 3 HORIZON trial, which is enrolling over 8000 patients with established cardiovascular disease and elevated Lp(a). These studies aim to confirm both the efficacy of Lp(a) lowering in reducing cardiovascular events and the long-term safety of pelacarsen, particularly in populations with significant comorbidities and advanced atherosclerotic disease. If successful, pelacarsen could represent a first-in-class therapy targeting Lp(a) and a major advance in the field of personalized cardiovascular prevention [114].

Currently registered active clinical trials involving Antisense Oligonucleotides, along with their objectives and interventions, are summarized in Table 5.

A summary of emerging therapies for lowering Lp(a) levels is presented in Table 6.

## 6. Current Guidelines and Recommendations

Given the growing evidence linking elevated Lp(a) to cardiovascular risk, several medical societies have incorporated Lp(a) into their clinical guidelines.

Recent clinical guidelines recognize Lp(a) as an independent and genetically determined risk factor for ASCVD. The 2021 recommendations from the European Society of Cardiology (ESC) and the European Atherosclerosis Society (EAS) advocate for a one-time measurement of Lp(a) in all adults, ideally during early adulthood. Levels exceeding 50 mg/dL (approximately 125 nmol/L) are considered elevated and associated with a higher risk of cardiovascular events. Although no Lp(a)-targeted therapies are currently approved, elevated concentrations are regarded as clinically relevant, particularly in individuals with premature ASCVD, familial hypercholesterolemia, or residual cardiovascular risk despite optimal lipid-lowering therapy. The guidelines emphasize the utility of Lp(a) assessment as a tool for refining cardiovascular risk stratification and guiding therapeutic intensity [126].

Similarly, the 2024 guidelines issued by the National Lipid Association (NLA), in collaboration with the American College of Cardiology (ACC) and the American Heart Association (AHA), classify elevated Lp(a) as a “risk-enhancing factor” in both primary and secondary prevention contexts. These recommendations propose Lp(a) testing in individuals with ambiguous cardiovascular risk profiles, a family history of premature ASCVD, or suboptimal response to lipid-lowering therapy. An Lp(a) concentration equal to or exceeding 50 mg/dL (125 nmol/L) is considered potentially actionable. While routine pharmacological reduction in Lp(a) is not currently recommended, elevated levels may warrant a more aggressive approach to cardiovascular risk management, including the consideration of adjunctive therapies such as PCSK9 inhibitors [127].

**Figure 3:**
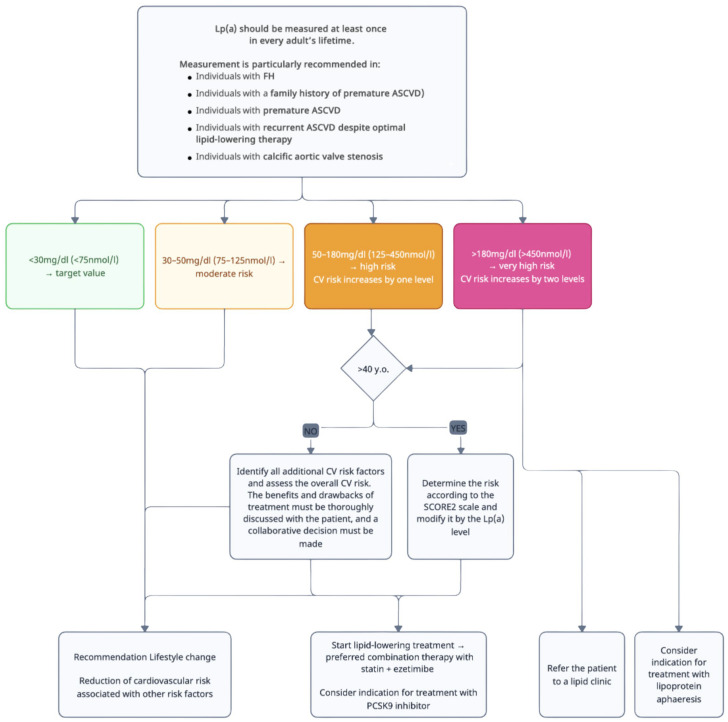
Decision tree based on Lp(a) concentration results. Own work.

Despite this increasing convergence in recognizing Lp(a) as a clinically meaningful risk modifier, practical implementation remains challenging. A range of position papers, expert consensuses, and evolving trial data offer recommendations that are partially overlapping and, at times, specific in nature. These recommendations address who to screen, how to interpret specific concentration thresholds, and how intensively to intervene. In routine clinical practice, this heterogeneity can impede the navigation of decision-making pathways, particularly as Lp(a) remains under-integrated into routine cardiovascular risk assessment and remains under-recognized by many clinicians. Consequently, the diagram presented below synthesizes available guidelines, consensus statements, and key evidence into a pragmatic framework outlining whom to test, how to stratify Lp(a) levels, their clinical implications, and potential management strategies. It is important to note that this scheme offers an adapted interpretation of current evidence, rather than an official or universally endorsed set of recommendations for clinical practice (Figure 3).

## 7. Conclusions

Elevated Lp(a) represents a well-established, independent risk factor for atherosclerotic cardiovascular disease, including myocardial infarction, stroke, and aortic valve stenosis. Despite its clinical relevance, Lp(a) has remained a challenging target due to its strong genetic determination and limited responsiveness to conventional lipid-lowering therapies. The emergence of novel agents—particularly antisense oligonucleotides, small interfering RNAs, and small-molecule inhibitors—has introduced a new era of targeted Lp(a)-lowering strategies, with early-phase trials demonstrating substantial efficacy and tolerability.

While these developments are encouraging, definitive evidence linking Lp(a) reduction to improved cardiovascular outcomes is still pending. Ongoing large-scale phase III trials will be instrumental in determining the clinical utility of these therapies. In the interim, Lp(a) measurement should be considered in selected high-risk populations, as recommended by current European and American guidelines, to enhance cardiovascular risk stratification and inform treatment decisions.

Future efforts should focus not only on validating therapeutic efficacy but also on refining patient selection and understanding the long-term safety of these novel interventions. Lp(a)-targeted therapy may soon become an integral component of precision cardiovascular medicine, provided that outcome-based evidence confirms its anticipated benefits.

## Figures and Tables

**Table 1:** Comparison of existing therapies for Lp(a).

Therapy	Mechanism of Action	Lp(a) Reduction & Clinical Relevance	Evidence Base	Impact on cv Outcomes	Key Limitations/Remarks
Statins	HMG-CoA reductase inhibition; indirect and on Lp(a) metabolism	−5% to +20% (statin-dependent); no clinically meaningful reduction;	Secondary analyses; not Lp(a)-targeted	Robust MACE reduction (LDL-C driven)	May increase Lp(a) in some patients
Ezetimibe	Inhibits intestinal cholesterol absorption via NPC1L1	0–7%; minimal and clinically negligible; heterogeneous findings	Small studies; inconsistent results	MACE reduction (LDL-C mediated)	Marginal and non-reproducible effect on Lp(a)
PCSK9 inhibitors	Inhibit PCSK9, preventing LDL receptor degradation and increasing receptor recycling	20–30%; consistent and potentially clinically relevant, though not directly proven	Large RCTs; Lp(a) assessed in secondary analyses	Proven MACE reduction; extent mediated by Lp(a) uncertain	Not primarily designed as Lp(a)-targeted therapy
Niacin	Inhibits adipocyte lipolysis; reduces hepatic VLDL and Lp(a) synthesis	20–40% (up to ~60% in selected cases); no demonstrated clinical benefit	RCT data available	No reduction in MACE despite Lp(a) lowering	Adverse effects; withdrawn in many regions
Lipoprotein apheresis	Extracorporeal mechanical removal of apoB-containing lipoproteins including Lp(a)	25–40% per session (transient effect); potentially relevant in extreme-risk patients	Observational studies; no large RCTs	Observational evidence suggests benefit	Invasive, costly, limited availability

**Table 3:** Clinical Trials investigating Lp(a) Inhibitors-based therapies for lowering Lp(a).

Aim of Trial	Conditions	Interventions	Control	Enrollment	Phases	Trial Status	NCT Number	Reference
Assess safety, side effects, and PK profile over 19–28 weeks	Healthy	Muvalaplin	Placebo	86	Phase 1	Completed	NCT04472676	[85]
Evaluate safety, PK, and tolerability over ~71 days	Healthy	Muvalaplin	Placebo	24	Phase 1	Completed	NCT05038787	[86]
Track excretion in urine, feces, air and compare oral vs. IV administration	Healthy	[^14^C]-Muvalaplin	-	16	Phase 1	Completed	NCT06342596	[87]
Compare PK and safety in renal impairment vs. normal renal function	Renal Insufficiency	Muvalaplin	-	47	Phase 1	Completed	NCT05778864	[88]
Evaluate efficacy and safety in patients with high Lp(a)	Lipoprotein Disorder	Muvalaplin	Placebo	233	Phase 2	Completed	NCT05563246	[89]
Evaluate the efficacy of muvalaplin in reducing cardiovascular risk in participants with high lipoprotein(a)	Elevated Lp(a) ASCVD	Muvalaplin	Placebo	10,450	Phase 3	Recruiting	NCT07157774	[84]

**Table 4:** Clinical Trials investigating CETP- based therapies for lowering Lp(a).

Aim of Trial	Conditions	Interventions	Control	Enrollment	Phases	Trial Status	NCT Number	Reference
Evaluate Lp(a) levels with obicetrapib ± evolocumab	Dyslipidemias	Obicetrapib Evolocumab	-	30	Phase 2	Recruiting	NCT06496243	[95]
Obicetrapib + ezetimibe in mild dyslipidemia: Assess efficacy/safety of combo therapy	Dyslipidemias High Cholesterol Hypercholesterolemia	Obicetrapib Ezetimibe	Placebo	112	Phase 2	Completed	NCT04770389	[96]
ApoB100 FCR with obicetrapib 10 mg: Determine effect on LDL apoB100 FCR	Lipid Metabolism	Obicetrapib	-	22	Phase 1	Active not recruiting	NCT05972278	[97]
Dose-finding with high-intensity statins: Test doses of obicetrapib with statins	Dyslipidemias High Cholesterol Hypercholesterolemia	Obicetrapib	-	120	Phase 2	Completed	NCT04753606	[98]
Food effect on fixed-dose obicetrapib/ezetimibe: Assess food impact on drug bioavailability	Healthy Volunteer Study	Fixed-Dose Combination (FDC) Tablet	-	28	Phase 1	Completed	NCT06050291	[99]
Interaction with drospirenone/ethinyl estradiol (COC): Evaluate PK interaction with COC components	Healthy Volunteers	Obicetrapib Drospirenone Ethinyl Estradiol	-	30	Phase 1	Completed	NCT06250205	[100]
Interaction with ezetimibe tablets: Study interaction with ezetimibe in healthy adults	Healthy Volunteers	Obicetrapib Ezetimibe	-	94	Phase 1	Completed	NCT06547359	[101]
Antioxidant impact in HDL, plasma, retina: Investigate antioxidant absorption with obicetrapib	Antioxidant Absorption	Obicetrapib	Placebo	100	Phase 2	Not yet recruiting	NCT06982508	[102]
Obicetrapib/ezetimibe in high-risk ASCVD (CCTA): Measure plaque/inflammation with CCTA	Lipidemia Coronary Artery Disease Plaque, ASCVD	Obicetrapib Ezetimibe	Placebo	300	Phase 3	Recruiting	NCT06305559	[103]
ASCVD not controlled on lipid therapy: Test obicetrapib in poorly controlled ASCVD	ASCVD	Obicetrapib	Placebo	9541	Phase 3	Active not recruiting	NCT05202509	[94]
HeFH/ASCVD or high-risk combo therapy: Evaluate combo therapy in HeFH or ASCVD risk	Dyslipidemias Hypercholesterolemia FA ASCVD	Obicetrapib Ezetimibe	Placebo	407	Phase 3	Completed	NCT06005597	[93]
Interaction with atorvastatin/rosuvastatin: Study PK interaction with statins	Healthy Volunteers	Obicetrapib Atorvastatin Calcium Rosuvastatin Calcium	-	74	Phase 1	Completed	NCT06081166	[104]
Obicetrapib in HeFH/ASCVD: Evaluate efficacy/safety in HeFH/ASCVD	Dyslipidemias High Cholesterol Hypercholesterolemia FA ASCVD	Obicetrapib	Placebo	2530	Phase 3	Completed	NCT05142722	[105]
PK/safety in moderate hepatic impairment: Compare PK in hepatic impairment vs. healthy	Hepatic Impairment Healthy	Obicetrapib	-	18	Phase 1	Completed	NCT06048302	[106]
Obicetrapib ± ezetimibe with statins: Evaluate mono vs. combo therapy in statin users	Dyslipidemias High Cholesterol Hypercholesterolemia	Obicetrapib Ezetimibe	Placebo	119	Phase 2	Completed	NCT05266586	[107]
Obicetrapib in HeFH patients: Assess obicetrapib in HeFH patients with a history	Dyslipidemias High Cholesterol FA Hypercholesterolemia Lipid Metabolism Disorder	Obicetrapib	Placebo	354	Phase 3	Completed	NCT05425745	[108]
Dose-finding in Japanese patients: Dose response in Japanese statin-treated patients	Dyslipidemia Hypercholesterolemia High Cholesterol	Obicetrapib	Placebo	102	Phase 2	Completed	NCT05421078	[109]
PK/PD/safety in early Alzheimer’s disease: PK/PD/safety in early Alzheimer’s population	Early Alzheimer’s Disease	Obicetrapib	-	13	Phase 2	Completed	NCT05161715	[110]

**Table 5:** Clinical trials investigating ASO- based therapies for lowering Lp(a).

Aim of Trial	Conditions	Intervention	Control	Enrollment	Study Phase	Trial status	NCT Number	Reference
List Managed Access Programs (MAPs) related to pelacarsen	ASCVD	Pelacarsen	-	-	N/A	Available	NCT07000695	[115]
Provide post-trial access to pelacarsen for participants of prior studies	ASCVD	Pelacarsen	-	600	Phase 3	Not yet recruiting	NCT06875973	[116]
Examine lipid subfractions, inflammation, and arterial wall properties post MI with elevated Lp(a)	Acute Coronary Syndrome Lipoproteinemia Inflammation Genetic Polymorphisms	Pelacarsen	Placebo	0	N/A	Withdrawn	NCT04993664	[117]
Provide access to pelacarsen in Germany for participants with hyperlipoproteinemia(a) and established CVD	Hyperlipoproteinemia (a)	Pelacarsen	-	41	Phase 3	Active not recruiting	NCT05900141	[118]
Support indication for cardiovascular risk reduction in CVD patients with elevated Lp(a)	Cardiovascular Disease and Lp(a)	Pelacarsen	Placebo	8323	Phase 3	Active not recruiting	NCT04023552	[119]
Evaluate Pelacarsen’s impact on ASCVD patients on inclisiran treatment for elevated LDL-C	ASCVD	Pelacarsen	Placebo Inclisiran	340	Phase 3	Not yet recruiting	NCT06813911	[120]
Evaluate PK, safety, and tolerability of Pelacarsen in healthy Japanese participants	Healthy Participants	Pelacarsen	Placebo	29	Phase 1	Completed	NCT05337878	[121]
Evaluate PK of Pelacarsen in mild hepatic impairment vs. healthy participants	Hepatic Impairment	Pelacarsen	-	17	Phase 1	Completed	NCT05026996	[122]
Evaluate efficacy of Pelacarsen for aortic valve stenosis progression: Evaluate efficacy of Pelacarsen in slowing calcific aortic valve stenosis progression	Aortic Stenosis	Pelacarsen	Matching placebo	502	Phase 2	Recruiting	NCT05646381	[123]
Evaluate Pelacarsen in US Black/Hispanic ASCVD participants to reduce cardiovascular risk	Elevated Lp(a) and ASCVD	Pelacarsen	Placebo	400	Phase 3	Recruiting	NCT06267560	[124]
Test Pelacarsen in reducing lipoprotein apheresis rate in hyperlipoproteinemia(a) patients	Hyperlipoproteinemia(a)	Pelacarsen	Corresponding Placebo	51	Phase 3	Completed	NCT05305664	[125]

**Table 6:** Comparison of emerging therapies for Lp(a).

Therapy	Mechanism of Action	Lp(a) Reduction Effectiveness	Route of Administration	Remarks
siRNA	Gene silencing via RNA interference, degrades Lp(a) mRNA	70–90%	Subcutaneous (injection)	Ongoing Phase 2/3 trials; promising efficacy and safety
ASO	Binds Lp(a) mRNA in liver, inhibits apo(a) synthesis	30–70%	Subcutaneous (injection)	Undergoing Phase 3 trials (HORIZON); favorable safety profile
Inhibitors of Lp(a) synthesis	Prevents apo(a) binding to apoB, blocks Lp(a) assembly	60–85%	Oral	Oral, promising for patient adherence; in clinical trials
CETP inhibitors	Inhibits CETP protein, reduces transfer of cholesterol esters	Up to 50%	Oral	Also lowers LDL-C; still under investigation for Lp(a)

## Data Availability

No new data were created or analyzed in this study.
